# 5-Bromo-*N* ^3^-[(*E*)-(6-bromo­pyridin-2-yl)methyl­idene]pyridine-3,4-diamine

**DOI:** 10.1107/S1600536811047702

**Published:** 2011-11-23

**Authors:** Mingjian Cai

**Affiliations:** a Department of Chemistry, Tangshan Normal University, Tangshan 063000, People’s Republic of China

## Abstract

The title compound, C_11_H_8_Br_2_N_4_, is a Schiff base obtained from 6-bromo­picolinaldehyde and 5-bromo­pyridine-3,4-diamine. The mol­ecule has an *E* configuration about the C=N bond and the dihedral angle between the two pyridine rings is 14.02 (1)°. The observed conformation is stabilised by an intramolecular N—H⋯N hydrogen bond. In the crystal, mol­ecules are stacked along the *b* axis and are linked through N—H⋯N hydrogen bonds into chains along the *c* axis.

## Related literature

For the use of Schiff bases in coordination, see: Burkhardt & Plass (2008 ▶); Keypour *et al.* (2011 ▶); Tarafder *et al.* (2002 ▶). For their properties, see: Kocyigit *et al.* (2010 ▶).
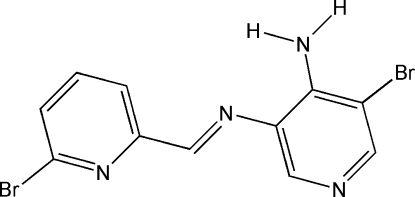


## Experimental

### 

#### Crystal data

C_11_H_8_Br_2_N_4_
*M* *_r_* = 356.03
Monoclinic, 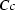

*a* = 24.941 (2) Å
*b* = 3.8306 (6) Å
*c* = 15.0868 (14) Å
β = 126.116 (14)°
*V* = 1164.4 (2) Å^3^
*Z* = 4
Mo *K*α radiation
μ = 6.94 mm^−1^
*T* = 113 K
0.20 × 0.18 × 0.12 mm

#### Data collection

Rigaku Saturn 724CCD diffractometer
Absorption correction: multi-scan (*CrystalClear*; Rigaku/MSC, 2002 ▶) *T*_min_ = 0.337, *T*_max_ = 0.490
5047 measured reflections
2282 independent reflections
2070 reflections with *I* > 2σ(*I*)
*R*_int_ = 0.046

#### Refinement

*R*[*F* ^2^ > 2σ(*F* ^2^)] = 0.029
*wR*(*F* ^2^) = 0.055
*S* = 0.89
2282 reflections
118 parameters
38 restraints
H-atom parameters constrained
Δρ_max_ = 0.45 e Å^−3^
Δρ_min_ = −0.63 e Å^−3^
Absolute structure: Flack (1983 ▶), 1093 Friedel pairs
Flack parameter: 0.002 (12)

### 

Data collection: *CrystalClear* (Rigaku/MSC, 2002 ▶); cell refinement: *CrystalClear*; data reduction: *CrystalClear*; program(s) used to solve structure: *SHELXS97* (Sheldrick, 2008 ▶); program(s) used to refine structure: *SHELXL97* (Sheldrick, 2008 ▶); molecular graphics: *DIAMOND* (Crystal Impact, 2009 ▶); software used to prepare material for publication: *CrystalStructure* (Rigaku/MSC, 2006 ▶).

## Supplementary Material

Crystal structure: contains datablock(s) I, global. DOI: 10.1107/S1600536811047702/ld2030sup1.cif

Structure factors: contains datablock(s) I. DOI: 10.1107/S1600536811047702/ld2030Isup2.hkl

Supplementary material file. DOI: 10.1107/S1600536811047702/ld2030Isup3.cml

Additional supplementary materials:  crystallographic information; 3D view; checkCIF report

## Figures and Tables

**Table 1:** Hydrogen-bond geometry (Å, °)

*D*—H⋯*A*	*D*—H	H⋯*A*	*D*⋯*A*	*D*—H⋯*A*
N4—H4*B*⋯N2	0.88	2.33	2.686 (6)	104
N4—H4*A*⋯N1^i^	0.88	2.44	3.043 (5)	126

Symmetry code: (i) 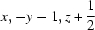.

**Fig. 1.**
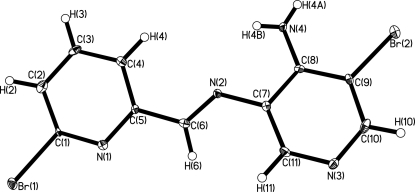
The molecular structure of the title compound, showing 30% probability displacement ellipsoids and the atom-numbering scheme.

**Fig. 2.**
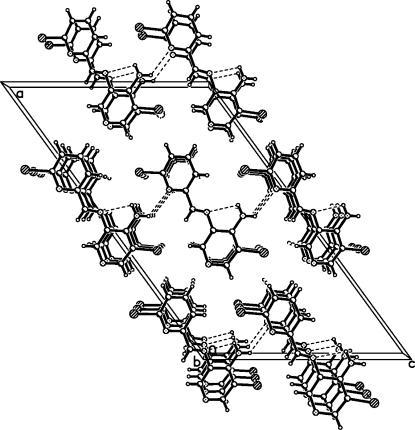
Molecular packing of the title compound with hydrogen bonding shown as dashed lines

## supplementary crystallographic information

### Comment

Schiff bases have played an important role in the development of coordination chemistry as they readily form stable complexes with most of the transition metals (Burkhardt & Plass, 2008; Keypour, *et al.*, 2011; Tarafder, *et al.*, 2002). They possess important properties, such as an ability to reversibly bind oxygen, catalytic activity in hydrogenation of olefins, transfer of an amino group, photochromic properties and complexing ability towards toxic metals (Kocyigit *et al.*, 2010). In this paper, a new Schiff base compound derived from condensation of 6-bromopicolinaldehyde with 5-bromopyridine-3,4-diamine is reported. The molecule of the title compound has an E configuration about the C6=N2 bond (Fig.1). The dihedral angle between the two pyridyl rings is 14.02 (1)°. An intramolecular N—H···N hydrogen bond forms five-membered ring. The five-membered ring and two pyridyl ring form dihedral angles of 3.60 (1)° and 4.02 (1)°. In the crystal, molecules are stacked along *y* axis and are linked through intermolecular N—H···N hydrogen bonds into chains propagating along *z* axis (Fig.2).

### Experimental

A solution of 6-bromopicolinaldehyde and 5-bromopyridine-3,4-diamine in methanol was refluxed for 30 min, and then the crude product was filtered and recrystallized from methanol to yield yellowish title compound. A small amount of the product was dissolved in methanol and the solution was kept for 5 days at ambient temperature to produce yellowish acicular crystals on slow evaporation of the solvent.

### Refinement

Amino H atoms were located in a difference fourier map and were put in ideal positions with N—H=0.88 Å. The remaining H atoms were positioned geometrically, with C—H=0.95 Å, and constrained to ride on their parent atoms, with *U*_iso_(H)=1.2*U*_eq_(C/N).

### Crystal data

**Table:**

C_11_H_8_Br_2_N_4_	*F*(000) = 688
*M**_r_* = 356.03	*D*_x_ = 2.031 Mg m^−^^3^
Monoclinic, *C**c*	Mo *K*α radiation, λ = 0.71073 Å
*a* = 24.941 (2) Å	Cell parameters from 2086 reflections
*b* = 3.8306 (6) Å	θ = 1.7–27.9°
*c* = 15.0868 (14) Å	µ = 6.94 mm^−^^1^
β = 126.116 (14)°	*T* = 113 K
*V* = 1164.4 (2) Å^3^	Prism, colorless
*Z* = 4	0.20 × 0.18 × 0.12 mm

### Data collection

**Table:**

Rigaku Saturn 724CCD diffractometer	2282 independent reflections
Radiation source: rotating anode	2070 reflections with *I* > 2σ(*I*)
multilayer	*R*_int_ = 0.046
Detector resolution: 14.22 pixels mm^-1^	θ_max_ = 26.4°, θ_min_ = 2.0°
ω scans	*h* = −30→29
Absorption correction: multi-scan (*CrystalClear*; Rigaku/MSC, 2002)	*k* = −4→4
*T*_min_ = 0.337, *T*_max_ = 0.490	*l* = −18→18
5047 measured reflections	

### Refinement

**Table:**

Refinement on *F*^2^	Hydrogen site location: inferred from neighbouring sites
Least-squares matrix: full	H-atom parameters constrained
*R*[*F*^2^ > 2σ(*F*^2^)] = 0.029	*w* = 1/[σ^2^(*F*_o_^2^) + (0.*P*)^2^] where *P* = (*F*_o_^2^ + 2*F*_c_^2^)/3
*wR*(*F*^2^) = 0.055	(Δ/σ)_max_ < 0.001
*S* = 0.89	Δρ_max_ = 0.45 e Å^−^^3^
2282 reflections	Δρ_min_ = −0.63 e Å^−^^3^
118 parameters	Extinction correction: *SHELXL97* (Sheldrick, 2008), Fc^*^=kFc[1+0.001xFc^2^λ^3^/sin(2θ)]^-1/4^
38 restraints	Extinction coefficient: 0.00177 (14)
Primary atom site location: structure-invariant direct methods	Absolute structure: Flack (1983), 1093 Friedel pairs
Secondary atom site location: difference Fourier map	Flack parameter: 0.002 (12)

### Special details

**Table:**

Geometry. All e.s.d.'s (except the e.s.d. in the dihedral angle between two l.s. planes) are estimated using the full covariance matrix. The cell e.s.d.'s are taken into account individually in the estimation of e.s.d.'s in distances, angles and torsion angles; correlations between e.s.d.'s in cell parameters are only used when they are defined by crystal symmetry. An approximate (isotropic) treatment of cell e.s.d.'s is used for estimating e.s.d.'s involving l.s. planes.
Refinement. Refinement of *F*^2^ against ALL reflections. The weighted *R*-factor *wR* and goodness of fit *S* are based on *F*^2^, conventional *R*-factors *R* are based on *F*, with *F* set to zero for negative *F*^2^. The threshold expression of *F*^2^ > σ(*F*^2^) is used only for calculating *R*-factors(gt)*etc*. and is not relevant to the choice of reflections for refinement. *R*-factors based on *F*^2^ are statistically about twice as large as those based on *F*, and *R*-factors based on ALL data will be even larger.

### Fractional atomic coordinates and isotropic or equivalent isotropic displacement parameters (Å^2^)

**Table:**

	*x*	*y*	*z*	*U*_iso_*/*U*_eq_	
Br1	−0.32411 (2)	−1.16292 (13)	−0.65216 (3)	0.01513 (13)	
Br2	−0.60799 (2)	0.03838 (13)	−0.34064 (3)	0.01849 (14)	
C6	−0.4590 (3)	−0.6690 (12)	−0.5248 (4)	0.0102 (6)	
H6	−0.4959	−0.7657	−0.5913	0.012*	
C1	−0.3284 (3)	−0.9595 (12)	−0.5398 (4)	0.0102 (7)	
C9	−0.5986 (3)	−0.1427 (11)	−0.4469 (4)	0.0102 (6)	
N2	−0.4692 (2)	−0.4839 (10)	−0.4673 (3)	0.0102 (6)	
C4	−0.3354 (3)	−0.6323 (13)	−0.3902 (4)	0.0117 (12)	
H4	−0.3393	−0.5114	−0.3393	0.014*	
C5	−0.3928 (3)	−0.7397 (13)	−0.4927 (4)	0.0102 (7)	
C8	−0.5367 (3)	−0.2611 (13)	−0.4152 (4)	0.0102 (6)	
N1	−0.3883 (2)	−0.9064 (10)	−0.5679 (3)	0.0102 (10)	
N3	−0.6504 (2)	−0.2897 (11)	−0.6358 (3)	0.0160 (11)	
C2	−0.2699 (3)	−0.8765 (12)	−0.4417 (4)	0.0102 (7)	
H2	−0.2282	−0.9341	−0.4266	0.012*	
C10	−0.6531 (3)	−0.1634 (12)	−0.5552 (4)	0.0102 (6)	
H10	−0.6947	−0.0846	−0.5738	0.012*	
N4	−0.4818 (2)	−0.2585 (10)	−0.3118 (3)	0.0156 (10)	
H4A	−0.4834	−0.1785	−0.2587	0.019*	
H4B	−0.4441	−0.3367	−0.2969	0.019*	
C3	−0.2743 (3)	−0.7040 (13)	−0.3652 (4)	0.0136 (12)	
H3	−0.2353	−0.6368	−0.2963	0.016*	
C11	−0.5907 (3)	−0.3985 (12)	−0.6050 (4)	0.0127 (13)	
H11	−0.5878	−0.4893	−0.6606	0.015*	
C7	−0.5329 (3)	−0.3906 (12)	−0.4994 (4)	0.0102 (6)	

### Atomic displacement parameters (Å^2^)

**Table:**

	*U*^11^	*U*^22^	*U*^33^	*U*^12^	*U*^13^	*U*^23^
Br1	0.0168 (3)	0.0139 (3)	0.0191 (3)	0.0022 (2)	0.0129 (2)	−0.0024 (2)
Br2	0.0207 (3)	0.0179 (3)	0.0219 (3)	0.0026 (3)	0.0153 (3)	−0.0004 (3)
C6	0.0118 (15)	0.0111 (14)	0.0086 (13)	−0.0005 (15)	0.0064 (12)	0.0002 (13)
C1	0.0100 (17)	0.0077 (15)	0.0135 (17)	−0.0007 (12)	0.0073 (14)	0.0026 (12)
C9	0.0122 (15)	0.0052 (13)	0.0154 (14)	−0.0006 (10)	0.0094 (12)	0.0015 (10)
N2	0.0118 (15)	0.0111 (14)	0.0086 (13)	−0.0005 (15)	0.0064 (12)	0.0002 (13)
C4	0.018 (3)	0.010 (3)	0.010 (3)	0.001 (2)	0.010 (3)	0.002 (2)
C5	0.0100 (17)	0.0077 (15)	0.0135 (17)	−0.0007 (12)	0.0073 (14)	0.0026 (12)
C8	0.0122 (15)	0.0052 (13)	0.0154 (14)	−0.0006 (10)	0.0094 (12)	0.0015 (10)
N1	0.013 (3)	0.008 (2)	0.008 (2)	−0.0011 (18)	0.006 (2)	0.0017 (17)
N3	0.009 (3)	0.023 (3)	0.012 (2)	0.003 (2)	0.004 (2)	0.0014 (19)
C2	0.0100 (17)	0.0077 (15)	0.0135 (17)	−0.0007 (12)	0.0073 (14)	0.0026 (12)
C10	0.0122 (15)	0.0052 (13)	0.0154 (14)	−0.0006 (10)	0.0094 (12)	0.0015 (10)
N4	0.005 (2)	0.031 (3)	0.010 (2)	0.0035 (19)	0.004 (2)	−0.0036 (18)
C3	0.008 (3)	0.017 (3)	0.008 (3)	−0.003 (2)	0.000 (2)	0.004 (2)
C11	0.016 (3)	0.011 (3)	0.012 (3)	0.002 (2)	0.009 (3)	0.000 (2)
C7	0.0122 (15)	0.0052 (13)	0.0154 (14)	−0.0006 (10)	0.0094 (12)	0.0015 (10)

### Geometric parameters (Å, °)

**Table:**

Br1—C1	1.925 (5)	C5—N1	1.363 (6)
Br2—C9	1.884 (5)	C8—N4	1.337 (6)
C6—N2	1.256 (5)	C8—C7	1.418 (7)
C6—C5	1.447 (7)	N3—C11	1.339 (6)
C6—H6	0.9500	N3—C10	1.347 (5)
C1—N1	1.305 (7)	C2—C3	1.389 (7)
C1—C2	1.370 (7)	C2—H2	0.9500
C9—C10	1.380 (6)	C10—H10	0.9500
C9—C8	1.397 (7)	N4—H4A	0.8800
N2—C7	1.407 (7)	N4—H4B	0.8800
C4—C3	1.363 (7)	C3—H3	0.9500
C4—C5	1.414 (7)	C11—C7	1.382 (7)
C4—H4	0.9500	C11—H11	0.9500
			
N2—C6—C5	122.0 (4)	C11—N3—C10	115.9 (5)
N2—C6—H6	119.0	C1—C2—C3	116.9 (5)
C5—C6—H6	119.0	C1—C2—H2	121.6
N1—C1—C2	127.0 (5)	C3—C2—H2	121.6
N1—C1—Br1	115.1 (4)	N3—C10—C9	123.4 (5)
C2—C1—Br1	117.9 (4)	N3—C10—H10	118.3
C10—C9—C8	120.4 (5)	C9—C10—H10	118.3
C10—C9—Br2	119.9 (4)	C8—N4—H4A	120.0
C8—C9—Br2	119.6 (4)	C8—N4—H4B	120.0
C6—N2—C7	123.5 (4)	H4A—N4—H4B	120.0
C3—C4—C5	119.4 (5)	C4—C3—C2	119.2 (5)
C3—C4—H4	120.3	C4—C3—H3	120.4
C5—C4—H4	120.3	C2—C3—H3	120.4
N1—C5—C4	121.3 (5)	N3—C11—C7	125.8 (5)
N1—C5—C6	116.6 (5)	N3—C11—H11	117.1
C4—C5—C6	122.1 (5)	C7—C11—H11	117.1
N4—C8—C9	124.2 (5)	C11—C7—N2	126.2 (5)
N4—C8—C7	119.0 (5)	C11—C7—C8	117.7 (5)
C9—C8—C7	116.8 (5)	N2—C7—C8	116.0 (5)
C1—N1—C5	116.2 (4)		
			
C5—C6—N2—C7	175.6 (5)	C11—N3—C10—C9	0.3 (7)
C3—C4—C5—N1	−1.5 (7)	C8—C9—C10—N3	−1.1 (7)
C3—C4—C5—C6	−179.4 (4)	Br2—C9—C10—N3	−180.0 (4)
N2—C6—C5—N1	−172.0 (4)	C5—C4—C3—C2	1.0 (7)
N2—C6—C5—C4	6.0 (7)	C1—C2—C3—C4	1.0 (7)
C10—C9—C8—N4	−178.3 (4)	C10—N3—C11—C7	−0.2 (8)
Br2—C9—C8—N4	0.6 (7)	N3—C11—C7—N2	−175.7 (5)
C10—C9—C8—C7	1.8 (7)	N3—C11—C7—C8	1.0 (8)
Br2—C9—C8—C7	−179.3 (3)	C6—N2—C7—C11	−19.0 (8)
C2—C1—N1—C5	2.4 (7)	C6—N2—C7—C8	164.2 (4)
Br1—C1—N1—C5	−175.4 (3)	N4—C8—C7—C11	178.4 (4)
C4—C5—N1—C1	−0.1 (7)	C9—C8—C7—C11	−1.7 (7)
C6—C5—N1—C1	177.9 (4)	N4—C8—C7—N2	−4.5 (7)
N1—C1—C2—C3	−2.9 (7)	C9—C8—C7—N2	175.4 (4)
Br1—C1—C2—C3	174.9 (3)		

### Hydrogen-bond geometry (Å, °)

**Table:**

*D*—H···*A*	*D*—H	H···*A*	*D*···*A*	*D*—H···*A*
N4—H4B···N2	0.88	2.33	2.686 (6)	104.
N4—H4A···N1^i^	0.88	2.44	3.043 (5)	126.

Symmetry codes: (i) *x*, −*y*−1, *z*+1/2.
